# Inflammation and Immune-Based Scores Predict Prognosis for Patients with Hepatocellular Carcinoma: A Pilot Single-Center Study

**DOI:** 10.5152/tjg.2025.24407

**Published:** 2025-01-06

**Authors:** Merve Guzel Dirim, Asli Cifcibasi Ormeci, Bilger Cavus, Arzu Poyanli, Kursat Rahmi Serin, Filiz Akyuz, Kadir Demir, Selman Fatih Besisik, Sabahattin Kaymakoglu

**Affiliations:** 1 Department of Internal Medicine, İstanbul University İstanbul Faculty of Medicine, İstanbul, Türkiye; 2 Division of Gastroenterohepatology, Department of Internal Medicine, İstanbul University İstanbul Faculty of Medicine, İstanbul, Türkiye; 3 Department of Radiology, İstanbul University İstanbul Faculty of Medicine, İstanbul, Türkiye; 4 Department of General Surgery, İstanbul University İstanbul Faculty of Medicine, İstanbul, Türkiye

**Keywords:** Hepatocellular carcinoma, C-reactive protein–albumin–lymphocyte index, alpha-fetoprotein, inflammation

## Abstract

**Background/Aims:**

Hepatocellular carcinoma (HCC) ranks as a major contributor to cancer-related deaths. Systemic inflammation plays a pivotal role in HCC development and progression. Thus, we aimed to determine the impact of inflammation- and immune-based scores in predicting the prognosis of HCC.

**Materials and Methods:**

In this retrospective study, patients with HCC were enrolled between 2010 and 2020. The purpose of this retrospective study was to evaluate the impact of various biomarkers, including baseline alpha-fetoprotein (AFP) levels, C-reactive protein (CRP)–albumin–lymphocyte (CALLY) index, neutrophil-to-CRP (N/CRP) ratio, systemic immune-inflammation index (SII), albumin–bilirubin (ALBI) score, neutrophil-to-lymphocyte ratio (NLR), and aspartate aminotransferase (AST)-to-alanine aminotransferase (ALT) (AST/ALT) ratio (AAR), on survival, invasion of vascular tracts, metastasis, and treatment responses.

**Results:**

A total of 199 patients with complete (n = 44) and non-complete (n = 145) treatment response groups were enrolled in the study. All scores for the non-complete response group were statistically significant (*P* < .05). The areas under the curves for predicting a non-complete response group were 0.651, 0.649, 0.636, 0.625, 0.613, 0.609, and 0.600 for AFP, CALLY index, AAR, SII, N/CRP ratio, ALBI score, and NLR, respectively. These results are consistent with the assessment of mortality and HCC progression.

**Conclusion:**

Our results indicate that these biomarkers could serve as powerful prognostic tools for HCC.

**Main Points**

- Systemic inflammation serves a crucial function in the development and progression of hepatocellular carcinoma. The assessment of prognostic scores based on inflammation and immunity is crucial for HCC follow-up.
- Liver function status is an additional parameter that can affect treatment decisions. Hepatocellular carcinoma development due to the progression of hepatic fibrosis in chronic liver disease is well known.
- In addition to alpha-fetoprotein, the CALLY index, SII, AAR, and ALBI scores are effective prognostic tools for HCC.

## Introduction

Hepatocellular carcinoma (HCC) is the most prevalent form of primary liver cancer and ranks among the leading causes of cancer-related mortality worldwide.^1^ Surgery, transplantation, locoregional treatment, and systemic treatment are the basis for HCC treatment.^2^ Despite the early initiation of treatment, recurrence and distant metastasis rates for HCC are reasonably high.^3^ Identifying patients with an increased risk of metastasis and recurrence is crucial for optimizing treatment. As such, it is essential to identify novel biomarkers that can aid in predicting outcomes and guiding treatment decisions.

Circulating tumor cells (CTCs) and their hematogenous spread play crucial roles in HCC metastasis. Neutrophils are the main factors involved in inflammation and adhesion to the bloodstream. According to several studies, neutrophilia is associated with poor prognosis by promoting immune-mediated tumor progression. In the bloodstream, neutrophils are the main factor for inflammation and adhesion.^4^ Platelets are another major factor in angiogenesis, cell proliferation, and tumor metastasis. However, lymphocytes are involved in the adaptive immune system and exert antitumor effects. The presence of tumor-infiltrating lymphocytes is associated with better survival in HCC.^5^ Taking into account these aspects, various prognostic scores have been created to predict survival and recurrence, including the neutrophil-to-lymphocyte ratio (NLR) and systemic immune-inflammation index (SII), both of which are based on inflammation and immune system factors.

Although serum albumin is a good marker of liver synthesis functions, some experimental studies have reported that it exerts protective effects by regulating tumor growth factors in HCC.^6^ The association of elevated C-reactive protein (CRP) levels before treatment with tumor progression and decreased liver function in HCC has been reported in the literature.^7^ Due to compromised immune systems, inadequate nutrition, and increased inflammation, the CRP–albumin–lymphocyte index (CALLY index) has been identified as a useful predictor for cancer, including those with HCC.

Liver function status is an additional parameter that can affect treatment decisions. In addition to the Child–Turcotte–Pugh (CTP) and MELD-Na scores, the albumin–bilirubin (ALBI) score predicts the reserve of liver function in HCC. Hepatocellular carcinoma development due to the progression of hepatic fibrosis is a well-known entity.^8^ The aspartate aminotransferase (AST)-to-alanine aminotransferase (ALT) ratio (AAR) has been validated as an index of hepatic fibrosis. Aspartate aminotransferase-to-alanine aminotransferase ratio is an independent indicator of aggressive progression in HCC.^9^ Alpha-fetoprotein (AFP) significantly reflects tumor biology, invasion, stage, and aggressiveness and is a significant predictor of post-transplant survival. According to previous research, high AFP values are associated with microvascular invasion and increased mortality.^10^ Numerous investigations have revealed a connection between these markers and the prognosis of individuals with HCC. Despite the limited availability of data, it is evident that these markers have not been adequately evaluated for efficacy in patients with HCC. Therefore, the objective of this study was to assess the impact of these scores on predicting the survival, recurrence, and mortality of HCC.

## Materials and Methods

We enrolled 199 patients with HCC at İstanbul University İstanbul Faculty of Medicine between 2010 and 2020. Hepatocellular carcinoma diagnosis was based on the criteria of the European Association for the Study of the Liver (EASL). This single-center retrospective study was approved by the İstanbul University İstanbul Faculty of Medicine Clinical Research Ethics Committee (approval number: 181150, date: 26.04.2021). Informed consent was obtained from all patients or their family members prior to their participation in the study. Patient information and records were de-identified and anonymized before being analyzed.

Background factors, including age, sex, presence or absence of comorbid diseases, history of cirrhosis or portal hypertension, and the etiology of chronic liver disease, were investigated. Tumor-related factors (maximum tumor size, total tumor diameter, number of tumor nodules, presence of vascular invasion, metastasis, and AFP level) were also evaluated. Considering the tumor burden and liver function status, the Barcelona Clinic Liver Cancer (BCLC) stage (0, A, B, C, D) was evaluated. The patients were stratified for liver transplantation according to the Milan and UCSF criteria. They were divided into subgroups (surgical resection, transplantation, transcatheter arterial chemoembolization (TACE), radiofrequency ablation (RFA), radioembolization, sorafenib, tyrosine kinase inhibitors, immunotherapy, and no treatment). The MELD-Na score and CTP classification were calculated based on blood counts and clinical parameters that were examined. The CALLY index was defined as (albumin × lymphocyte)/(CRP × 10^4^). We also calculated other scoring systems, including NLR, neutrophil-to-CRP (N/CRP) ratio, AAR, and SII. The ALBI score was calculated using the following formula: ALBI score = (log10 bilirubin × 0.66) + (albumin × −0.085), where the units of bilirubin and albumin were µmol/L and g/L, respectively. Patients were then classified into 3 grades based on the ALBI score: grade I, score ≤ −2.60; grade II, score −2.60 to ≤ −1.39; and grade III, score > −1.39.

The primary endpoint of the study was defined as the absence of HCC after treatment in the last outpatient or inpatient control. According to the primary endpoint, patients were categorized as having a complete treatment response or a non-complete treatment response (partial response, stable disease, and progressive disease). The secondary endpoint was the mortality rate. We evaluated the impact of baseline AFP, SII, CALLY index, N/CRP ratio, NLR, ALBI score, and AAR on vascular thrombosis, extrahepatic spread, and primary and secondary endpoints. The analyses of univariate and multivariate logistic regression were conducted to identify the independent factors associated with mortality. Furthermore, the area under the curve (AUC) of the receiver operating characteristic (ROC) curves was calculated to evaluate the predictive performance.

### Statistical Analysis

The statistical analysis was carried out using the SPSS 26 software package (IBM SPSS Corp.; Armonk, NY, USA). The normality of the quantitative data was evaluated using the Shapiro–Wilk test. The Mann–Whitney *U*-test was used to compare the quantitative variables between 2 groups that did not follow a normal distribution. The Kruskal–Wallis test and Dunn–Bonferroni test were used for the comparison of more than 2 quantitative variables that did not follow a normal distribution. The Pearson chi-square test, Fisher’s exact test, and Fisher-Freeman-Halton test were used to compare qualitative data. Spearman’s correlation analysis was used to evaluate the relationship between quantitative variables. The cutoff values for continuous variables were determined using ROC curve analysis. The factors that were significant in the univariate analysis (*P* < .05) were included in the multivariate analysis using a Cox forward stepwise variable selection process for the estimated mortality.

## Results

In the initial cohort, there were 199 individuals, comprising 76.9% men and 23.1% women. The mean age at the time of enrollment was 63 years. The etiologies for HCC were hepatitis-B virus (56.2%) and hepatitis-C virus (24.6%), followed by metabolic dysfunction-associated steatohepatitis (MASH) (9%). About 8.5% of the patients had an unclassified etiology. Liver cirrhosis and portal hypertension were observed in 177 (88.8%) and 127 (63.8%) patients, respectively. The majority of the cohort had CTP A (69.3) with a mean MELD-Na score of 10.2. The patient’s characteristics according to BCLC Stage and treatment characteristics are shown in Table 1. High pretreatment AFP levels (>400 ng/mL) were observed in 32 (17.2%) patients. The median tumor size was 3 cm, and 32 patients had ≥ 3 tumors. There were 45 (22.6%) and 154 (77.4%) patients in the complete- and non-complete-response groups, respectively. The mean follow-up period was 56.9 ± 4.5 months.

### Association of the Biomarkers with Prognosis and Clinicopathologic Parameters

The prognostic significance of the biomarkers with pretreatment total tumor size, BCLC stage, and suitability for liver transplantation according to the UCSF and Milan criteria was analyzed. Higher levels of AFP, NLR, AAR, ALBI score, SII, and decreased CALLY index and N/CRP ratio were associated with increased pretreatment total tumor size, advanced BCLC stage, and out-of-UCSF criteria (*P* < .05). A similar relationship was found for the Milan criteria, except for ALBI score. Increased levels of baseline AFP (*P*= .002), NLR (*P*= .043), AAR (*P*= .006), ALBI score (*P*= .027), SII (*P*= .012), and lower CALLY index (*P*= .005) and N/CRP ratio (*P*= .034) were prognostic impacts for non-complete treatment response for HCC. Odds ratios (OR) for the variables in the same order were OR: 5.018, 95% CI: 2.108-11.947; OR: 6.753, 95% CI: 2.266-20.124; OR: 2.948, 95% CI: 1.461-5.948; OR: 2.405, 95% CI: 1.204-4.806; OR: 2.979, 95% CI: 1.270-6.990; OR: 2.675, 95% CI: 1.342-5.331; OR: 2.494 95% CI: 1.193-5.211.

**Figure 1.**
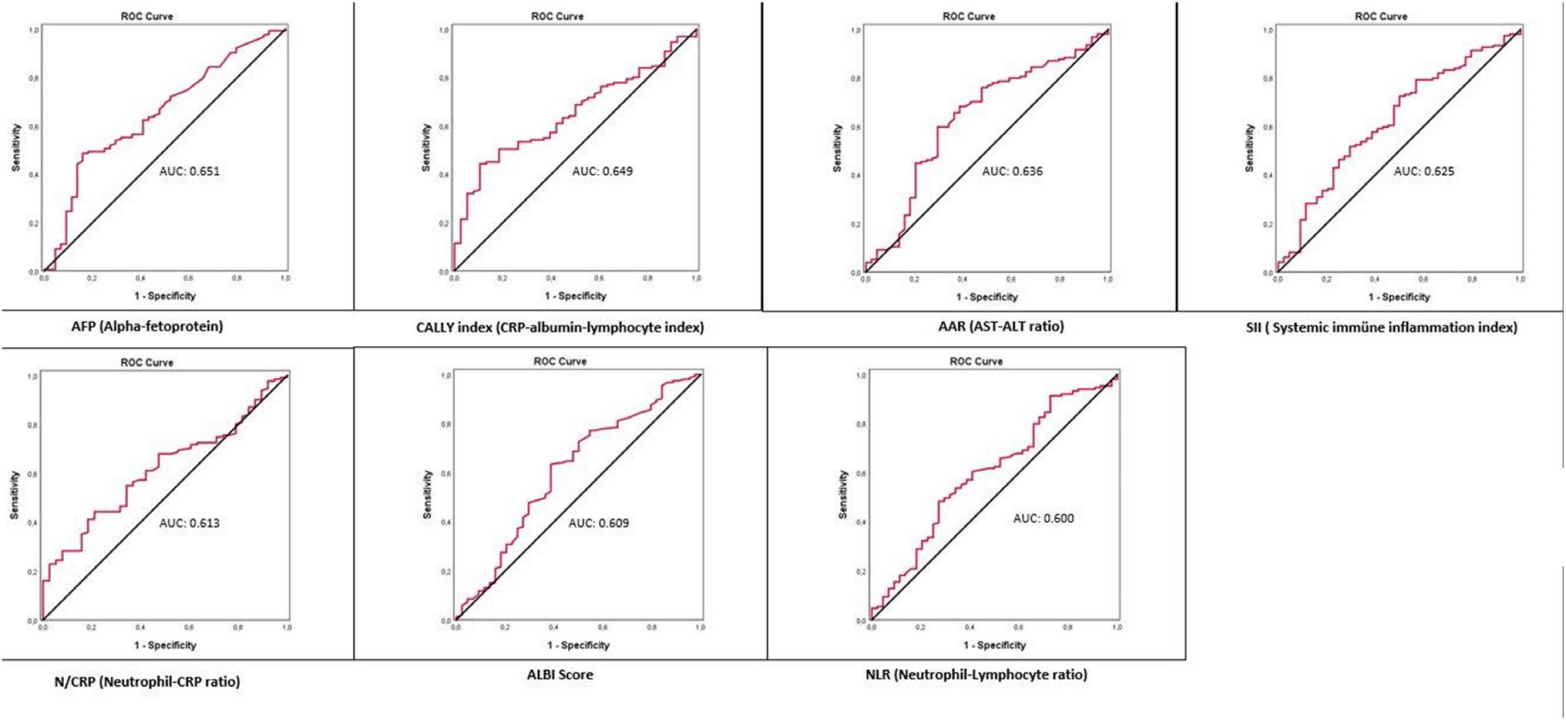
Receiver operating characteristic analyses with non-complete treatment response for prognostic markers.

The discriminatory ability of the prognostic scores and clinical indices was compared using the AUC for non-complete treatment responses (Figure 1). The AUC for AFP was 0.651 (95% CI: 2.108-11.947), which was the most potent biomarker among the indices for predicting a lack of response to treatment. The CALLY index was the second most important biomarker, almost equally efficient with AFP (AUC: 0.649, 95% CI: 0.561-0.737). Aspartate aminotransferase-to-alanine aminotransferase ratio was the other significant score after AFP; however, NLR had the lowest impact on prognostic value (Table 2).

Consistent with previous analyses, higher levels of pretreatment AFP, NLR, AAR, ALBI score, SII, and decreased CALLY index and N/CRP ratio were associated with the presence of vascular invasion and last tumor size (Table 3). A similar correlation was observed for extrahepatic spread. Nevertheless, there was no significant association between metastasis, ALBI score, and NLR (*P*: .226, *P*= .183).

A subgroup analysis of 97 patients who were transplant candidates within the MILAN criteria was conducted to evaluate clinicopathological parameters. An elevated AFP level was found to be associated with vascular invasion (*P*= .038), while an increase in the ALBI score was associated with extrahepatic spread (*P=* .012). No other biomarkers demonstrated prognostic significance.

Viral hepatitis and MASH, as 2 primary etiological factors for HCC, were included in the subgroup analysis. Elevated levels of baseline AFP, NLR, AAR, ALBI score, SII, and decreased CALLY index and N/CRP ratio were associated with vascular invasion and final tumor size in viral hepatitis (Table 4). However, no significant association was observed with metastasis. In MASH, only a significant relationship was found between increased AAR and ALBI scores and vascular invasion (*P*= .03, *P*= .025).

### Survival Analysis

After a mean follow-up of 56.9 ± 4.5 months, 121 patients (61.8%) died, and the mean survival rate was detected as 61.94 ± 5.14 months. In the multivariate analysis, the ALBI score (HR; 2.361 [1.74-3.21], *P*= .001), NLR (HR; 1.11 [1.037-1.188], *P*= .003), AFP level (HR; 1 [1-1], *P*: .045), BCLC stage C (HR; 4.993 [1.918-12.997], *P*= .001), and BCLC stage D (HR; 25.435 [4.719-137.09], *P*= .001) were found to be significant (Table 5). Patient age, chronic liver disease duration, and the presence of other comorbid diseases were not associated with survival. Child–Turcotte–Pugh grade was not an independent risk factor for mortality after adjusting for other factors in the multivariate analysis.

## Discussion

To the best of our knowledge, this is the first study to investigate and compare the predictive ability of multiple scores for HCC treatment response. Numerous studies have revealed the prognostic significance of the CALLY index and the percentage of neutrophils in HCC.^4,11,12^ Neutrophils might play a regulatory role in the tumor microenvironment due to their role in the myeloid suppressor cell spectrum. Various prognostic models based on serum CRP and albumin levels have been developed to demonstrate the role of systemic inflammation in malignancies.^13,14^ The significance of assessing CRP levels lies in their capacity to serve as an unbiased indicator of systemic inflammation. The association between elevated CRP levels before treatment and tumor progression and decreased liver function in HCC has been reported in the literature. The higher CRP/albumin ratio was associated with tumor progression, high CLIP and BCLC scores, and decreased liver function reserve.^15^

In our study, lower pre-treatment N/CRP ratio and CALLY index were found to be the determining factors in the non-complete treatment response group, with an increase in final tumor diameter, vascular invasion, metastasis, and mortality. Receiver operating characteristic analyses were performed among the N/CRP ratio and the CALLY index, and the efficiency of the CALLY index was higher (AUC 0.649; 95% CI 0.613). This superiority can be explained by the fact that the CALLY index provides more information about the immunonutritive status of HCC by including parameters that determine inflammation, host immune response, and nutritional status. Previous studies have identified the CALLY index as a significant biomarker for certain treatment groups, such as hepatectomy or TACE.^16^ To the best of our knowledge, this is the first study to reveal the efficiency of the CALLY index in a Turkish population of patients with HCC.

The liver function reserve plays a crucial role in the resection or treatment decisions of HCC. Albumin level is a good indicator of liver synthesis. In addition, some experimental studies have shown that albumin exerts protective effects by regulating tumor growth factors in HCC. Because albumin synthesized in differentiated hepatocytes, normal serum albumin levels indicate well-differentiated and slow-growing HCC biology.^17^ Furthermore, the albümin level is a prognostic factor for nutritional status. The ALBI score and grade have been developed using serum albumin and bilirubin levels.^18^ Unlike the CTP score, the ALBI score is not based on subjective evaluations. Dividing the patients in CTP A as ALBI grades I and II could show an advantage for patients with early-stage HCC. Survival analyses of patients with primary curative resection showed that patients with ALBI grade I had better survival.^19^ In our study, a higher baseline ALBI score was associated with increased tumor size, advanced BCLC stage, and out-of-UCSF HCC criteria.

It is worth noting that HCC frequently develops in cirrhotic liver parenchyma. The AAR is a validated indicator of the histological degree of liver fibrosis and cirrhosis. Wang et al^9^ reported that an increase in AAR was associated with portal vein invasion and early recurrence in patients who underwent curative HCC resection. The increased AAR was also found to be more effective in predicting HCC than liver cirrhosis. Liu et al^20^ demonstrated an association between higher baseline AAR and worse survival and treatment failure in patients experiencing transarterial chemoembolization. In our study, a higher AAR at the time of diagnosis was associated with increased tumor size, advanced BCLC stage, out-of-UCSF, and Milan criteria. The results showed that baseline AAR was associated with worse outcomes, such as increased tumor size, increased vascular invasion, and metastasis development in the last control.

Serum inflammatory markers are crucial for evaluating survival and treatment success in solid tumors and lymphomas. It is stated in the literature that circulating neutrophil levels may be associated with a worse prognosis by causing immune-related tumor progression and cancer cachexia syndrome compared to platelets and lymphocytes.^21^ However, cytotoxic CD8 lymphocytes play a crucial role in antitumor activity. The presence of tumor-infiltrating lymphocytes in HCC resection materials is associated with better survival outcomes.^5,22^ For these reasons, a high NLR represents increased protumoral activity and decreased antitumor immune function.^23^

Recent studies have shown that increased SII levels are associated with liver cirrhosis, vascular invasion in HCC, a high BCLC stage, and early recurrence.^24^ Hong et al^25^ determined that increased SII levels were associated with a worse prognosis in HCC patients receiving sorafenib and regorafenib treatment. In our study, increased SII and NLR levels were correlated with the last tumor size, advanced BCLC stage, out-of-UCSF, and Milan criteria, and increased mortality. Similarly, an increase in SII levels was detected in the non-complete treatment response group, and baseline SII values were associated with increased tumor size, increased vascular invasion, and metastasis development. Although NLR showed similar results in the non-complete treatment response group, it was not associated with extrahepatic spread. Based on these results, SII better reflects the balance between the patient’s inflammation and immune response. In our study, NLR had the weakest prognostic impact among all the parameters (AUC: 0.600; *P*= .043).

As serum AFP has proven to be a dependable biomarker for the diagnosis of HCC, pre-diagnostic screening programs, and the assessment of survival, we assessed AFP using other inflammation-based and immunonutritional scoring systems. Increased AFP levels at the time of diagnosis had significant importance for non-complete treatment response, last tumor diameter, vascular invasion, increased metastasis, and mortality. Receiver operating characteristic analyses revealed that AFP ≥63 ng/mL values were associated with worse survival outcomes in our study. In EASL guidelines, AFP significantly reflects tumor biology, invasion, stage, and aggression and is a revealing marker in predicting post-transplant survival.^26^ Furthermore, high AFP (≥200 ng/mL) is associated with increased microvascular invasion and high mortality in patients with HCC receiving sorafenib treatment. The results obtained in our study are also consistent with the existing literature data.^27,28^

The objective of this study was to evaluate the predictive value of biomarkers for chronic inflammation, fibrosis, and immunonutrition in patients with HCC. Receiver operating characteristic analyses demonstrated that AFP, CALLY Index, and AAR were the most effective scoring systems in predicting non-complete treatment response and patient prognosis. While AFP is a direct marker of tumor biology and remains superior to other inflammation-based biomarkers, it is known that elevated AFP levels may not be present during HCC diagnosis. In our study, nearly half of the patients had low AFP levels (<20 ng/dL), which is consistent with previous studies in the literature.^29^ Due to this limitation of AFP during diagnosis and follow-up, other scoring systems have become noteworthy. The CALLY index was the second-most significant prognostic scoring system, combining inflammation, immune response, and nutritional status markers as additional components of a comprehensive patient evaluation. In the future, promising stratification and scoring systems could be developed by combining the CALLY index with other immunonutritive parameters such as sarcopenia. Consequently, this index can enhance patient stratification and enable individualized prognostic predictions.

In our research, we assessed the ALBI score in the CTP A-dominant patient population, which aligns with the existing literature. This might serve as a substitute for evaluating liver function reserve. However, further scientific data are necessary for HCC and advanced liver failure. In conjunction with the biomarkers, our survival analyses incorporated factors such as age, duration of chronic liver disease, presence of comorbidities, total tumor diameter, and BCLC stage. In the multivariate analysis, the ALBI score, NLR, AFP level, BCLC stage C, and BCLC stage D were found to be significant. No difference was observed in the distribution of the ALBI grade. Those with BCLC stages C and D were determined to be more influential than those with stages 0, A, and B. The prominent role of models based on liver function and tumor stage in the multivariate analysis suggests that mortality in HCC is primarily due to complications arising from chronic liver disease and tumor progression.

Viral hepatitis is the primary reason for hepatic carcinogenesis, although MASH is emerging as a significant etiological factor in chronic liver disease. Glycemic dysregulation and obesity are the principal metabolic factors that contribute to an oxidative hepatic environment conducive to HCC pathogenesis. In viral hepatitis, immune-mediated liver injury, extensive fibrosis, and cirrhosis contribute to HCC development. Furthermore, viral hepatitis promotes carcinogenesis through viral proteins that stimulate oncogene expression, deactivate tumor suppressor genes, and disrupt cell cycle regulation. In a subgroup analysis of etiological factors of HCC, our research demonstrated that all scores are associated with vascular invasion and final tumor size; however, they are not correlated with extrahepatic spread in patients with viral hepatitis. However, only a weak association was observed between elevated AAR and ALBI scores and vascular invasion in patients with MASH. The median follow-up duration was similar for both etiologies (120 months). Although MASH-related inflammation can lead to fibrosis and subsequent HCC, the progression may be slower or occur in conjunction with other metabolic conditions. In addition to causing liver fibrosis and cirrhosis, virus-related direct oncogenic activity through its structural proteins constitutes an additional risk factor for HCC pathogenesis.^30,31^

This study had some limitations. The number of patients was relatively small, and the majority of the cohort comprised CTP class A patients. The patient characteristics varied in terms of BCLC stages and treatment modalities. Although this patient cohort may not have focused on specific treatment groups, it comprehensively revealed the clinical characteristics and outcomes of HCC. Thus, this study provides valuable real-life data that can guide the clinical follow-up of HCC in the Turkish population. The formulas used in the study were simple and allowed for easy calculations. Additionally, our study indicated that the CALLY index, AAR, SII, and ALBI score may serve as potential tools for predicting HCC prognosis. However, further prospective and multicenter studies with larger sample sizes are required to validate these findings.

In conclusion, the CALLY index, AAR, SII, and ALBI score show promise as predictive biomarkers for evaluating treatment response in HCC. To our knowledge, this is the initial study to evaluate the comparison between AFP and other immune- and inflammation-based prognostic scores in patients with HCC. Considering their practicality and simplicity, these biomarkers should be considered for the management of HCC.

## Figures and Tables

**Table 1.** Demographic and Baseline Characteristics of Patients

Age (years)	Mean ± SD	62.77 ± 11.05
	Median (min-max)	63 (50-90)
Gender (%)	Male	153 (76.9)
	Female	46 (23.1)
Etiology (%)	Idiopathic	11 (5.5)
	HBV	112 (56.2)
	HCV	49 (24.6)
	Alcohol	3 (1.5)
	MASH Autoimmune hepatitis Primary biliary cholangitis Budd–Chiari	18 (9.0) 2 (1.0) 2 (1.0) 2 (1.0)
Child–Pugh (%)	A	138 (69.3)
	B	44 (22.1)
	C	17 (8.5)
MELD-Na	Mean ± SD Median	10.27 ± 4.17 9 (6-27)
BCLC stage (%)	0 A B C D	20 (3.7) 57 (10.4) 68 (12.5) 48 (8.8) 6 (1.1)
Liver fibrosis (%)	Normal	22 (11.1)
	Cirrhosis	177 (88.8)
Portal hypertension (%)		127 (63.8)
HCC diagnosis (%)	Biopsy MRI	15 (7.5) 184 (92.5)
Median total tumor size (cm)	Min-max	3 (0.9-44)
Tumor number, n (%)	Unifocal Multifocal	120 (60.9) 79 (39.1)
Treatment methods, n (%)	Surgical resection Transplantation TACE Radioembolization RFA Sorafenib Regorafenib	4 (2) 19 (9) 38 (19) 7 (3) 16 (8) 82 (41) 6 (3)
AFP—baseline (ng/dL)	Mean ± SD	5076.27 ± 25725.97
	<20	86 (43.4)
	20-400	64 (32.3)
	401-10 000	34 (17.2)
	>10 000	14 (7.1)

AFP, alpha-fetoprotein; HBV, hepatitis-B virus; HCC, hepatocellular carcinoma; HCV, hepatitis-C virus; MASH, metabolic dysfunction-associated steatohepatitis; MELD-Na, model for end-stage liver disease-sodium; RFA, radiofrequency ablation; TACE, transcatheter arterial chemoembolization.

**Table 2.** Discrimination Ability of Prognostic Scores for Non-Complete Treatment Response

	Diagnostic Scan					ROC Curve		*P*
	Cut off	Sensitivity	Specificity	Positive Predictive Value	Negative Predictive Value	Area	95% CI	
AFP	≥63	48.7	84.09	91.5	31.9	**0.651**	0.560-0.742	**.002**
NLR	≥2.98	48.32	72.73	85.7	29.4	**0.600**	0.504-0.697	**.043**
N/CRP	≤357.14	44.27	78.95	87.9	29.1	**0.613**	0.522-0.705	**.034**
AST/ALT	≥1.37	59.74	70.45	87.6	33.3	**0.636**	0.540-0.732	**.006**
ALBI score	≥-2.60	60.78	61.36	84.5	31	**0.609**	0.510-0.708	**.027**
CALLY index	≤0.41	44.27	89.47	93.5	31.8	**0.649**	0.561-0.737	**.005**
SII	≥210.91	72.48	50	83.1	34.9	**0.625**	0.531-0.718	**.012**

AAR, AST/ALT ratio; AFP, alpha-fetoprotein; BCLC, Barcelona clinic liver cancer; CALLY index, CRP–albumin–lymphocyte index; N/CRP, neutrophil-to-CRP ratio; NLR, neutrophil-to-lymphocyte ratio; SII, systemic immune-inflammation index.

**Table 3.** Prognostic Factors of Treatment Response and Clinicopathological Parameters

	Treatment Response			Vascular Invasion	Metastasis	Last Tumor Size	
	Complete Response (n)	Non-Complete Response (n)	*P*	*P*	*P*	*r*	*P*
AFP	44	154	**.002^c^**	**.001^c^**	**.004^c^**	0.27	**.001^d^**
NLR	44	149	**.043^c^**	**.001^c^**	.183^c^	0.258	**.001^d^**
N/ CRP	38	131	**.034^c^**	**.001^c^**	**.001^c^**	−0.361	**.001^d^**
AST/ALT	44	154	**.006^c^**	**.001^c^**	**.001^c^**	0.284	**.001^d^**
ALBI score	44	153	**.027^c^**	**.001^c^**	.226^c^	0.185	**.016^d^**
CALLY index	38	131	**.005^c^**	**.001^c^**	**.002^c^**	−0.413	**.001^d^**
SII	44	149	**.012^c^**	**.005^c^**	**.051^c^**	0.265	**.001^d^**

*Bold values indicate statistical significance at the P<0.05 level ; r* = Spearman’s correlation coefficient.

AAR, AST/ALT ratio; AFP, alpha-fetoprotein; CALLY index, CRP–albumin–lymphocyte index; NLR, neutrophil-to-lymphocyte ratio; N/CRP, neutrophil-to-CRP ratio; SII, systemic immune-inflammation index.

^c^Mann–Whitney *U*-test.

^d^Spearman Correlation test.

**Table 4.** Prognostic Factors of Clinicopathological Parameters in Patients with Viral Hepatitis and Metabolic Dysfunction-Associated Steatohepatitis

Viral Hepatitis					MASH			
	Vascular Invasion	Metastasis	Last Tumor Size		Vascular Invasion	Metastasis	Last Tumor Size	
	*P*	*P*	*r*	*P*	*P*	*P*	*r*	*P*
AFP	**.000^c^**	.144^c^	0.245	**.005**	.468^c^	.295^c^	0.338	.238
NLR	**.000^c^**	.232^c^	0.298	**.001**	.563^c^	.297^c^	0.237	.414
N/ CRP	**.000^c^**	.282^c^	−0.295	**.001**	.194^c^	.487^c^	−0.35	.22
AST/ALT	**.029^c^**	.228^c^	0.259	**.003**	**.030^c^**	.908^c^	0.186	.525
ALBI score	**.000^c^**	.701^c^	0.218	**.013**	**.025^c^**	.862^c^	0.045	.879
CALLY index	**.000^c^**	.055^c^	−0.413	**.000**	.061^c^	.908^c^	−0.232	.424
SII	**.001^c^**	.026^c^	0.292	**.001**	.470^c^	.355^c^	0.336	.241

*r* = Spearman’s correlation coefficient.

AAR, AST/ALT ratio; AFP, alpha-fetoprotein; CALLY Index, CRP–albumin–lymphocyte index; MASH, metabolic dysfunction-associated steatohepatitis; N/CRP, neutrophil-to-CRP ratio; NLR, neutrophil-to-lymphocyte ratio; SII, systemic immune-inflammation index. .

^c^Mann–Whitney *U*-test.

**Table 5.** Prognostic Factors for Mortality in the Patient Cohort

	Univariate Analysis		Multivariate Analysis	
	HR (95% CI)	*P*	HR (95% CI)	*P*
AFP	1 (1-1)	.169	1 (1-1)	**.045**
NLR	1.194 (1.13-1.27)	**.001**	1.110 (1.037-1.188)	**.003**
N/CRP	1000 (0.999-1)	**.002**		
AAR	1.742 (1.313-2.310)	**.001**		
ALBI score	2.012 (1.57-2.58)	.001**	2.361 (1.74-3.21)	.001**
ALBI grade				
I	Reference			**.001**
II	2.814 (1.89-4.20)	**.001**	2.968 (1.844-4.775)	**.001**
III	3.46 (1.84-6.48)	**.001**	5.839 (2.476-13.770)	**.001**
CALLY index	0.769 (0.67-0.88)	**.001**		
SII	1.001 (1.00-1.001)	**.001**		
Age	1.003 (0.986-1.020)	.735		
Chronic liver disease duration (months)	1.001 (0.999-1.003)	.272		
Total tumor size	1.040 (1.021-1.061)	**.001**	1.029 (0.999-1.060)	.055
Comorbid disease	0.705 (0.493-1.007)	.055		
Child–Pugh classification				
A	Reference			
B	1.867 (1.232-2.829)	**.003**		
C	3.810 (2.099-6.916)	**.001**		
BCLC stage				
Stage 0	Reference		Reference	
Stage A	1.382 (0.601-3.176)	.446	1.374 (0.536-3.517)	.508
Stage B	2.148 (0.965-4.780)	.061	2.328 (0.919-5.895)	.075
Stage C	5.908 (2.609-13.377)	**.001**	4.993 (1.918-12.997)	**.001**
Stage D	29.543 (9.486-92.01)	**.001**	25.435 (4.719-137.094)	**.001**

AAR, AST/ALT ratio; AFP, alpha-fetoprotein; BCLC, Barcelona Clinic Liver Cancer; CALLY index, CRP–albumin–lymphocyte index; N/CRP, neutrophil-to-CRP ratio; NLR, neutrophil-to-lymphocyte ratio; SII, systemic immune-inflammation index.

## Data Availability

The datasets during and/or analyzed during the current study are available from the corresponding author upon reasonable request.
